# Supplementary material for: Association of Delirium and Depression with Respiratory and Outcome Measures in COVID-19 Inpatients
Source: J Pers Med. 2023 Jul 29;13(8):1207. doi: 10.3390/jpm13081207 (PMC10456095; doi:10.3390/jpm13081207)
Supplement: Supplementary file 1 [file jpm-13-01207-s001.zip › jpm-2361743-supplementary.pdf]

Suppl. Table S1. Predictors for levels of oxygen partial pressure (PaO_2) in participants with depressive symptoms (DEP)

Predictors	Univariate analyses					Multivariate analyses				
	R^2	β	*t*	95% CI	*p*	R^2	β	*t*	95% CI	*p*
Psychopathological										
HAM-D	.12	-.38	-2.55	-1.70, -.19	**.015**	.34	-.28	-2.03	-1.39, -.002	**.041**
HAM-A	.01	-.17	-1.10	-1.08, .32	.280					
YMRS	-.02	.07	.44	-1.31, 2.05	.660					
BPRS	-.01	-.12	-.76	-1.21, .55	.452					
Clinical										
Age	.02	-.10	-.61	-.36, .19	.543					
Female	-.02	.13	.82	-4.70, 11.13	.416					
Employed	.01	-.17	-1.08	-12.13, 3.67	.285					
Live alone	.06	.29	1.87	-.57, 15.50	.068					
Education	-.02	-.05	-.33	-1.13, .81	.739					
Comorbidities	-.02	-.25	-.22	-2.54, .2.03	.825					
BMI	.01	-.18	-1.06	-1.18, .37	.299					
Pneumonia	.01	.19	1.19	-4.18, 15.88	.245					
Dyspnea	.14	-.40	-2.71	-18.77, -2.72	**.010**	.34	-3.27	-2.41	-16.22, -1.41	**.021**
Hyperthermia	-.03	-.04	-.24	-9.14, 7.22	.813					
Cough	-.02	-.06	-.35	-10.22, 7.25	.732					
Drug treatments prior to admission	.11	-.37	-2.51	-3.46, -.35	**.017**	.34	-2.87	-2.11	-1.39, -.002	**.049**
CFS	-.03	-.05	-.28	-2.62, 1.99	.783					
ADL	-.01	-.18	-1.15	-3.12, .86	.257					
IADL	-.004	-.14	-.89	-2.16, .84	.378					

Note: Significant *p*-values ($p<0.05$) are indicated in **bold**. DEP: participants with depressive symptoms. ADL: activities of daily living scale; BMI, body mass index; BPRS, Brief Psychiatric Rating Scale, CFS; clinical frailty scale; HAM-A, Hamilton Anxiety Rating Scale; HAM-D, Hamilton Depression Rating Scale, IADL, instrumental activities of daily living scale; PaO_2, oxygen partial pressure; SD, standard deviation; YMRS, Young Mania Rating Scale.

Suppl. Table S2. Predictors for levels of ratio of arterial oxygen partial pressure to fractional inspired oxygen (PaO_2/FiO_2) in participants with depressive symptoms (DEP)

Predictors	Univariate analyses					Multivariate analyses				
	R^2	β	*t*	95% CI	*p*	R^2	β	*t*	95% CI	*P*
HAM-D	0.17	-.43	-2.99	-10.50, -2.02	**.005**	.32	-4.04	-2.80	-10.92, -.26	**.012**
HAM-A	.08	-.32	-2.07	-7.92, -0.94	**.045**	.32	-.171	-1.41	-5.31, .956	.167
YMRS	-.03	-.01	-.03	-9.60, 9.32	.976					
BPRS	-.006	-.14	-.87	-8.23, 3.30	.392					
Age	.04	-.25	-1.64	-2.78, .29	.109					
Female	-.012	.12	.73	-29.31, 62.35	.470					
Employed	-.015	.10	.64	-31.46, 60.78	.524					
Live alone	.04	.25	1.62	-9.27, 84.65	.113					
Education	-.02	-.06	-.38	-4.52, 6.63	.704					
Comorbidities	.04	-.25	-1.63	-23.10, 2.50	.112					
BMI	.02	-.22	-1.34	-7.28, 1.50	.189					
Pneumonia	-.02	.08	.495	-44.37, 73.06	.624					
Dyspnea	.31	-.57	-4.35	-130.76, -47.78	**<.001**	.32	-.49	-4.37	-112.95, -41.44	**<.001**
Hyperthermia	-.03	.03	.17	-43.05, 51.09	.864					
Cough	-.02	.06	.36	-41.41, 59.10	.724					
Drug treatments prior to admission	.15	-.41	-2.82	-21.12, -3.51	**.007**	.32	-.31	-2.72	-16.08 -2.36	**.010**
CFS	.05	-.18	-1.68	-23.34, 2.17	.101					
ADL	.01	-.20	-1.16	-18.12, 4.90	.252					
IADL	-.01	-.11	-.66	-11.57, 5.88	.513					

Note: Significant p-values ($p<0.05$) are indicated in bold. DEP: participants with depressive symptom. ADL: activities of daily living scale; BMI, body mass index; BPRS, Brief Psychiatric Rating Scale, CFS; clinical frailty scale; HAM-A, Hamilton Anxiety Rating Scale; HAM-D, Hamilton Depression Rating Scale, IADL, instrumental activities of daily living scale; PaO_2/FiO_2, arterial oxygen partial pressure to fractional inspired oxygen; YMRS, Young Mania Rating Scale. SD, standard deviation.

Suppl. Table S3. Predictors for duration of hospitalization in participants with depressive symptoms (DEP)

Predictors	Univariate analyses					Multivariate analyses				
	R^2	β	*t*	95% CI	*p*	R^2	β	*t*	95% CI	*p*
HAM-D	-.03	.03	.18	-.66, .79	.857					
HAM-A	.02	-.21	-1.33	-1.03, .21	.191					
YMRS	-.03	-.04	-.22	-1.68. 1.36	.829					
BPRS	.16	.43	2.98	.35, 1.83	**.005**	.52	.31	2.78	.22, 1.37	**.008**
Age	.04	.25	1.63	-.05, .44	.112					
Female	-.01	.11	.70	-4.65, 9.60	.486					
Employed	.08	-.32	-2.13	-13.99, -.35	**.040**	.52	-.186	-1.67	.-9.26, .88	.103
Live alone	.02	-.07	-.47	-9.32, 5.82	.643					
Education	-.03	.01	.06	-.80, .85	.956					
Comorbidities	.01	.13	-.844	-1.19, 2.90	.404					
BMI	.01	-1.20	.19	-1.18, .31	.241					
Pneumonia	-.03	.04	.22	-8.06, 10.06	.824					
Dyspnea	-.03	.02	.10	-7.40, 8.14	.924					
Hyperthermia	.004	.17	1.08	-3.33, 10.96	.287					
Cough	-.03	.002	.01	-7.72, 7.79	.993					
Drug treatments prior to admission	-.02	.06	.41	-1.20., 1.80	.686					
CFS	-.002	.16	.96	-1.07, 2.99	.344					
ADL	.39	-.63	-5.20	-5.07, -2.23	**<.001**	.49	-.59	-5.22	-4.67, -2.06	**<.001**
IADL	-.29	-.55	-4.19	-3.52, -1.23	**<.001**					

Note: Significant *p*-values ($p<0.05$) are indicated in **bold**. DEP: participants with depressive symptoms. ADL: activities of daily living scale; BMI, body mass index; BPRS, Brief Psychiatric Rating Scale, CFS; clinical frailty scale; HAM-A, Hamilton Anxiety Rating Scale; HAM-D, Hamilton Depression Rating Scale, IADL, instrumental activities of daily living scale; YMRS, Young Mania Rating Scale. SD, standard deviation.

Suppl. Table S4. Predictors for levels of oxygen partial pressure (PaO_2) in participants with delirium (DEL).

Predictors	Univariate analyses					Multivariate analyses				
	R^2	β	*t*	95% CI	*p*	R^2	β	*t*	95% CI	*p*
NEECHAM	.35	.59	3.45	.88, 3.53	**.002**	.62	.56	4.11	1.02, 3.12	**.001**
Age	-0.15	-.17	-.82	-.797, .346	.422					
Female	-0.41	.07	.32	-12.08, 16.51	.751					
Employed	-043	.05	-24	-12.44, 15.67	.814					
Live alone	-.05	-.08	-.36	-18.83, 13.35	.725					
Education	-.055	-.03	-.12	-2.19, 1.96	.910					
Comorbidities	-.01	-.17	-.83	-1.53, 3.55	.417					
BMI	-.06	-01	.06	-1.78, 1.88	.954					
Pneumonia	-.01	-.19	-.89	-24.00, 9.57	.382					
Dyspnea	.04	-.28	-1.35	-22.35, 4.69	.189					
Hyperthermia	.28	-.56	-3.15	-29.12, -6.01	**.005**	.62	-.52	-3.83	-25.18, -7.46	**.008**
Cough	-.02	-.15	-.71	-19.56, 9.54	.483					
Drug treatments prior to admission	-.013	.18	.84	-1.58, 3.72	.411					
CFS	-.05	-.03	-.15	-3.60, 3.13	.886					
ADL	-.04	-.08	-.35	-3.81, 2.72	.730					
IADL	-.02	-.16	-.74	-3.01, 1.43	.467					

Note: Significant *p*-values ($p<0.05$) are indicated in **bold**. DEL: participants with delirium. ADL: activities of daily living scale; BMI, body mass index; CFS; Clinical Frailty Scale; IADL, instrumental activities of daily living scale; NEECHAM, Neelson and Champagn Confusion Scale; PaO_2, oxygen partial pressure. SD, standard deviation.

Suppl. Table S5. Predictors for levels of ratio of arterial oxygen partial pressure to fractional inspired oxygen (PaO_2/FiO_2) in participants with delirium (DEL)

Predictors	Univariate analyses					Multivariate analyses				
	R^2	β	*t*	95% CI	*p*	R^2	β	*T*	95% CI	*p*
NEECHAM	.34	.61	3.58	5.62, 21.12	**.002**	.61	.60	4.55	7.16, 19.31	**<.001**
Age	-.03	-.14	-.64	-4.46, 2.35	.526					
Female	-.04	.03	.16	-78.33, 91.22	.876					
Employed	-.04	-.06	.27	-72.44, 93.87	.792					
Living alone	-.02	-.17	-.74	-127.55, 60.70	.466					
Education	.084	-.36	-1.65	-20.37, 2.43	.115					
Comorbidities	.04	.29	1.42	-4.58, 24.62	.169					
BMI	-.02	.19	.80	-7.03, 15.68	.434					
Pneumonia	.04	-.28	-1.39	-161.83, 32.10	.179					
Dyspnea	.15	-.44	-2.26	-156.88, -6.84	**.034**	.61	-.27	-1.64	-116.47, 13.84	**.**116
Hyperthermia	.26	-.54	-2.99	-169.72, -30.76	**.007**	.61	-.330	1.98	-125.92, 3.08	.061.
Cough	-.04	.07	.33	-73.21, 100.59	.747					
Drug treatments prior to admission	.02	.25	1.19	-6.61, 24.24	.248					
CFS	-.03	.14	.64	-13.98, 26.37	.529					
ADL	-.04	-.12	-.54	-24.77, 14.62	.597					
IADL	.02	-.26	-1.22	-20.82, 5.47	.237					

Note: Significant *p*-values ($p<0.05$) are indicated in **bold**. DEL: participants with delirium. ADL: activities of daily living scale; BMI, body mass index; CFS; Clinical Frailty Scale; IADL, instrumental activities of daily living scale; NEECHAM, Neelson And Champagn Confusion Scale; PaO_2/FiO_2, ratio of arterial oxygen partial pressure to fractional inspired oxygen. SD, standard deviation.
